# Potential Relationship Between Decreased Serum Selenium Levels and Oxidative Stress in Periodontitis Stage III-IV

**DOI:** 10.1007/s12011-025-04649-3

**Published:** 2025-05-05

**Authors:** Mehmetcan Uytun, Recep Orbak, Ahmet Kızıltunç

**Affiliations:** 1 Department of Periodontology, School of Dentistry, Muğla Sıtkı Koçman University, Muğla, 48000 Turkey; 2 Department of Periodontology, School of Dentistry, Atatürk University, Erzurum, 25100 Turkey; 3 Department of Medical Biochemistry, School of Medicine, Atatürk University, Erzurum, 25100 Turkey

**Keywords:** Antioxidants, Free radicals, Oxidative stress, Periodontitis, Selenium, Trace elements

## Abstract

In this study, the relationships between periodontitis and total oxidant status (TOS), total antioxidant status (TAS), and selenium levels were investigated. A total of 122 participants, including 61 periodontitis patients and 61 periodontally healthy individuals, were included. Serum TOS, TAS, and selenium levels were measured, and the biochemical and clinical parameters were compared. The relationship between selenium levels and periodontitis was assessed through univariate analysis and multivariate logistic regression. Compared with the healthy group, the periodontitis group had significantly higher TOS and significantly lower TAS and selenium levels (*p* < 0.001). Logistic regression analysis also revealed a significant correlation between selenium levels and periodontitis (*p* < 0.001). Our study demonstrated that periodontitis was related to TOS, TAS, and selenium levels. The present study investigated the relationships of periodontitis with TOS, TAS, and selenium levels. Selenium levels could serve as an important biomarker for periodontal disease, as they are strongly correlated with the TOS value, the TAS value, and clinical parameters. Furthermore, lower selenium levels were observed in periodontitis patients than in healthy individuals.

## Introduction

Periodontitis is an inflammatory disease that affects tooth-supporting tissues and degrades connective tissue, periodontal ligaments, and alveolar bone [1]. Studies have demonstrated that while periodontopathogens initiate periodontitis, actual tissue loss is caused primarily by the host response [2]. In periodontitis patients, the initial host response to pathogens involves polymorphonuclear leukocytes (PMNLs), which perform phagocytosis and utilize oxidative processes to produce reactive oxygen species (ROS), thereby eliminating pathogens [3].

ROS are short-lived and highly reactive by-products of oxygen metabolism in living tissues. PMNL activation increases the production of free radicals (FRs), including superoxide and hydroxyl radicals. Previous studies have reported higher levels of superoxide radicals in periodontitis patients than in healthy individuals [4, 5]. ROS cause tissue damage through various mechanisms, including DNA damage, lipid peroxidation, protein damage, and enzyme oxidation [3]. They disrupt collagen cross-linking, increasing susceptibility to collagenase [6], and are involved in osteoclast production, regulation, and alveolar osteonecrosis [7]. In periodontitis, the ROS generated by PMNLs cause oxidative damage to mitochondrial DNA, potentially exacerbating oxidative stress due to the downregulation of proteins involved in electron transport and cell death [8]. Living organisms have evolved antioxidant systems to counteract these detrimental effects of ROS [9].

The antioxidant defence system that protects against the detrimental effects of FRs involves antioxidant molecules that capture, stabilize, or slow the production of FRs. It also prevents, reduces, or delays ROS-induced damage [10]. On the basis of these functions, organisms utilize antioxidant defence systems to maintain tissue health [9, 11, 12]. Normally, there is a balance between oxidants and antioxidants in the human body, but increased ROS production or decreased antioxidant levels can disrupt this balance [3], leading to a type of cellular damage known as oxidative stress. Previous studies have reported that oxidative stress can lead to various inflammatory and collagen tissue diseases, including periodontitis [9, 13–15]. Trace elements, such as zinc, copper, and selenium, are necessary for the action of antioxidant enzymes, contributing to the preservation of periodontal tissue health against the destructive effects of oxidative stress [16].

Selenium is a trace element essential for the activity of antioxidant enzymes [17]. It plays a crucial role in preventing the damage caused by ROS because of its potent antioxidant properties, ability to support the normal cell cycle, ability to maintain cell viability, and ability to regulate enzyme activity [18]. Organic selenium exists in the form of selenocysteine or selenomethionine. Selenocysteine, an amino acid structurally similar to cysteine but containing selenium instead of sulfur [19], is incorporated into selenoproteins, which require selenium for their function. Thirty selenoproteins have been identified in the human body [20], and they are involved in the biological effects of selenium. Selenium and selenoproteins contribute to immune regulation, reducing the risk of chronic inflammation and autoimmunity [21]. Selenium, in the form of selenocysteine, is also a component of important antioxidant enzymes, such as glutathione peroxidase. Among glutathione peroxidases, selenium has been reported to mitigate the harmful effects of hydrogen peroxide (H_2_O_2_) and lipid peroxides, thereby reducing ROS levels [16, 22]. Previous studies have demonstrated the involvement of selenium in a complex antioxidant defence system against oxidative stress, which is mediated by selenium-dependent glutathione peroxidase or selenoproteins [23].

Previous studies have reported increased oxidative stress biomarker levels and decreased total antioxidant levels in periodontitis patients [24, 25]. However, the relationships between periodontitis and total oxidant status (TOS), total antioxidant status (TAS), and selenium levels have not been evaluated in previous studies. Therefore, serum TOS values, TAS values, and selenium levels were assessed in both periodontitis patients and healthy individuals and the relationships between these biochemical parameters and clinical periodontal parameters were investigated. Our hypothesis was that periodontitis is associated with serum TOS values, TAS values, and selenium levels and that selenium levels could serve as a diagnostic parameter for periodontitis activity.

## Materials and Methods

### Study Design and Participants

The study involved 122 volunteers who applied to the Ataturk University Faculty of Dentistry between June 2021 and October 2021, including 57 females and 65 males ranging in age from 22–49 years. Using G*Power software version 3.1 (Heinrich-Heine-University, Düsseldorf, Germany), the required sample size was calculated on the basis of data from a reference study. To detect a significant difference with 80% power and a 95% confidence interval, it was estimated that 110 participants would be needed for inclusion in the study [23]. However, considering the potential for sample loss, a total of 122 participants were included.

Ethical approval was obtained from the Ataturk University Faculty of Medicine Clinical Research Ethics Committee on 15 April 2021 (decision number: 03/53). The study was conducted in accordance with the Declaration of Helsinki.

The inclusion criteria were individuals aged between 18 and 65 years who had not undergone periodontal treatment within the past year; had not used any medications within the previous six months; and had not taken vitamin, mineral, or antioxidant supplements within the past three months. The exclusion criteria included individuals who smoked or consumed alcohol, those who were pregnant or lactating, and individuals with any chronic or systemic diseases. All participants provided consent on forms detailing the study methodology.

### Study Groups

The study groups were divided into two groups, periodontitis and periodontally healthy, on the basis of the 2017 classification developed through a collaboration between the European Federation of Periodontology and the American Academy of Periodontology [26]. The periodontitis group (P) included 61 individuals diagnosed with stage III or IV periodontitis. The inclusion criteria for this group were individuals who had ≥ 15 teeth and exhibited clinical attachment loss (CAL) of ≥ 5 mm in ≥ 1 tooth, pocket depth (PD) of ≥ 6 mm in ≥ 1 tooth, and radiographic bone loss extending along the middle or apical third of the root. The periodontally healthy group (H) comprised 61 periodontally healthy individuals. The inclusion criteria for this group were individuals with ≥ 15 teeth who exhibited a CAL of ≤ 1 mm in all teeth, PD of ≤ 3 mm in all teeth, absence of radiographic bone loss, and < 10% bleeding on probing (BOP) in the oral cavity.

### Clinical Evaluation

Clinical and radiographic examinations were conducted to assess the periodontal status of the participants. The clinical evaluation included the plaque index (PI), gingival index (GI), PD, and CAL measurements for all teeth [27–29].

These measurements were taken by expert periodontologists (M.U. and R.O.). Before the study, calibration was completed, with 10 patients diagnosed with stage III or IV periodontitis. Intra- and inter-examiner reliability were evaluated using the intraclass correlation coefficient (ICC). The intra-examiner ICCs were 0.88 (PD) and 0.89 (CAL) for M.U. and 0.90 (PD) and 0.92 (CAL) for R.O. The inter-examiner values were 0.89 (PD) and 0.88 (CAL). Consequently, the reliability of the measurements was considered satisfactory.

### Serum Sample Acquisition

After an overnight fasting period, 8 mL of venous blood was aseptically collected from the antecubital vein of each participant using a sterile, disposable syringe. The blood samples were drawn into yellow-capped vacuum tubes with separation gel (BD Vacutainer® SST™ II Advance, Chicago, IL, USA). Prior to centrifugation, the tubes were left at room temperature for 15 min and subsequently centrifuged in a refrigerated centrifuge at 3,500 rpm for 10 min at 4 °C to obtain serum (NÜVE NF1200R, Ankara, Türkiye). The obtained serum was carefully transferred into Eppendorf tubes and stored at − 80 °C until analysis. The samples were thawed at + 4 °C approximately 24 h prior to analysis.

### Oxidative Evaluation

TOS was measured using a commercial assay kit (Product ID: MH16068O; Rel Assay Diagnostics, Gaziantep, Türkiye). In accordance with the manufacturer’s guidelines, the results of these measurements were recorded in μmol/L. TAS measurements were performed using another commercial total antioxidant status assay kit (Product ID: MH16059 A; Rel Assay Diagnostics, Gaziantep, Türkiye). In accordance with the manufacturer’s instructions, the results of these measurements were recorded in mmol/L.

### Selenium Analysis

Selenium concentrations were determined following a previously described protocol with minor modifications to the sample preparation procedure [30]. In brief, 0.1 mL of serum was transferred into 15 mL Teflon tubes, followed by the addition of 0.5 mL of a concentrated HNO₃/H₂O₂ (4:1) solution. The samples were subsequently digested using a closed-vessel microwave digestion system (MILESTONE Ethos Easy Advanced Microwave Digestion System, Italy) at 100 °C for 105 min. After the digestion procedure, the final volume of each sample was adjusted to 10 mL with ultrapure water. Selenium levels were quantified using inductively coupled plasma–mass spectrometry (ICP-MS, 7700 Series; Agilent Technologies, Santa Clara, CA, USA). Calibration standards for selenium were prepared in increasing concentrations of 2% HNO₃ solution, and calibration curves were constructed accordingly. The ICP‒MS instrument was operated under optimized conditions with a radio frequency power of 1550 W, a helium (He) flow rate of 4.3 mL/min, and an argon (Ar) plasma gas flow rate of 15 L/min. The auxiliary and carrier gas flow rates were set at 1.0 L/min and 0.99 L/min, respectively. The selenium concentrations obtained from the samples were recorded in μg/L.

### Statistical Analysis

Statistical analyses were conducted using IBM SPSS 20 (IBM Corp., Armonk, NY, USA). The data are presented as the means, standard deviations, medians, minimum and maximum values, percentages, and numbers. The normality of the distribution of continuous variables was assessed using the Shapiro–Wilk and Kolmogorov–Smirnov tests. The independent samples *t* test was used for analysing normally distributed data, whereas the Mann–Whitney U test was used for comparisons of nonnormally distributed data between groups. For categorical variables, Pearson’s chi-square test was used if the expected value was > 5, the chi-square Yates test was used if it ranged between 3 and 5, and Fisher’s exact test was used if it was < 3 in 2 × 2 comparisons. For comparisons between two quantitative variables, the Pearson correlation test was used for normally distributed data; otherwise, the Spearman correlation test was used. Analysis of covariance (ANCOVA) was used to analyse the effects of cofactors on the dependent variable in multiple comparisons. The statistical significance level was set at *p* < 0.05.

## Results

### Demographic Parameters

**Fig. 1:**
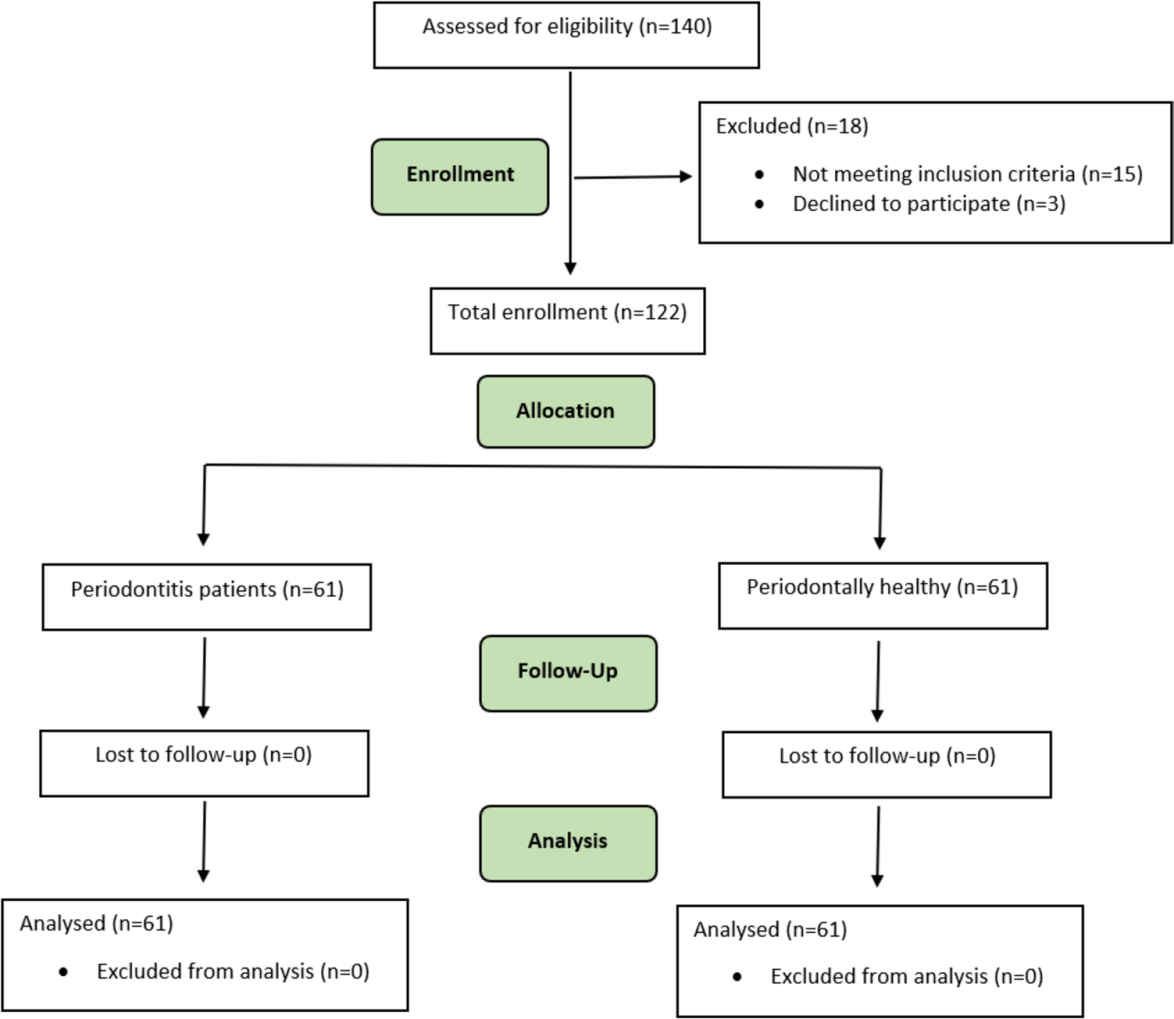
Flow chart of study

Among the 140 individuals initially examined, 122 who met the criteria were included in our study, comprising 57 females and 65 males. Three patients refused to participate, and fifteen did not meet the criteria, resulting in their exclusion from the study (Fig. 1). The demographic information of the participants in the study groups is presented in Table 1.

**Table 1:** Demographics of the study groups

	Periodontitis (*N* = 61)	Healthy (*N* = 61)		*p* value
Age				
Mean ± sd	36.20 ± 6.84	30.95 ± 4.53	Z = −5.060	< 0.001^*^
Mdn.(min.-max.)	36 (22–49)	30 (26–49)		
Sex				
Female	28	29	X^2^ = 0.033	0.856
Male	33	32		

Values are presented as mean ± standard deviation (sd), median (Mdn.), minimum (min.), maximum (max.)

Z: Mann Whitney U test, X^2^: Chi-squared test

*p < 0,05 and p-values less than 0.05 are indicated in bold

### Clinical Parameters

**Fig. 2:**
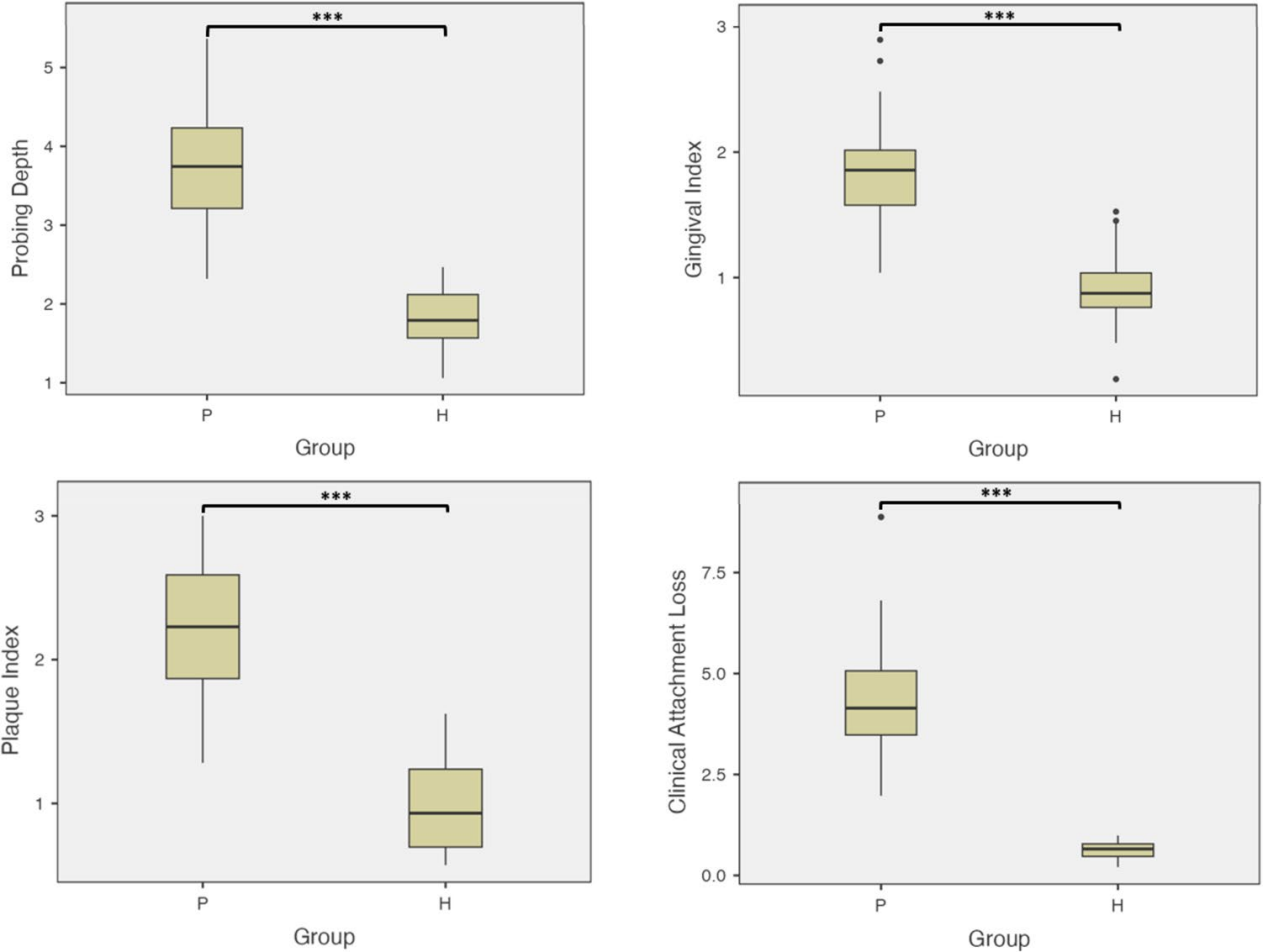
Boxplots of clinical parameters. All clinical parameters were significantly higher in Group P compared to Group H (*p* < 0.001). *** indicates statistical significance between groups (*p* < 0.001). P: Periodontitis group; H: Periodontally healthy group

All the clinical parameters (PI, GI, PD, and CAL) were significantly higher in Group P than in Group H (*p* < 0.001) (Fig. 2). Group comparisons of the PI, GI, PD, and CAL values are presented in Table 2.

**Table 2:** Group comparisons in terms of clinical parameters

	Periodontitis (*N* = 61)	Healthy (*N* = 61)		*p* value
PI				
Mean ± sd	2.20 ± 0.46	0.98 ± 0.30	Z = −9.286	< 0.001^*^
Mdn.(min.-max.)	2.23(1.28–3.00)	0.93(0.57–1.62)		
GI				
Mean ± sd	1.81 ± 0.39	0.91 ± 0.26	t = 15.128	< 0.001^*^
Mdn.(min.-max.)	1.86(1.04–2.90)	0.87(0.19–1.53)		
PD(mm)				
Mean ± sd	3.77 ± 0.72	1.80 ± 0.37	t = 18.958	< 0.001^*^
Mdn.(min.-max.)	3.74(2.32–5.37)	1.79(1.06–2.47)		
CAL(mm)				
Mean ± sd	4.37 ± 1.19	0.64 ± 0.21	t = 24.197	< 0.001^*^
Mdn.(min.-max.)	4.14(1.97–8.88)	0.65(0.21–0.99)		

Values are presented as mean ± standard deviation (sd), median (Mdn.), minimum (min.), maximum (max.)

Z: Mann Whitney U test, t: Independent samples t test

*p < 0,05 and *p*-values less than 0.05 are indicated in bold

### TOS, TAS and Selenium Levels

**Fig. 3:**
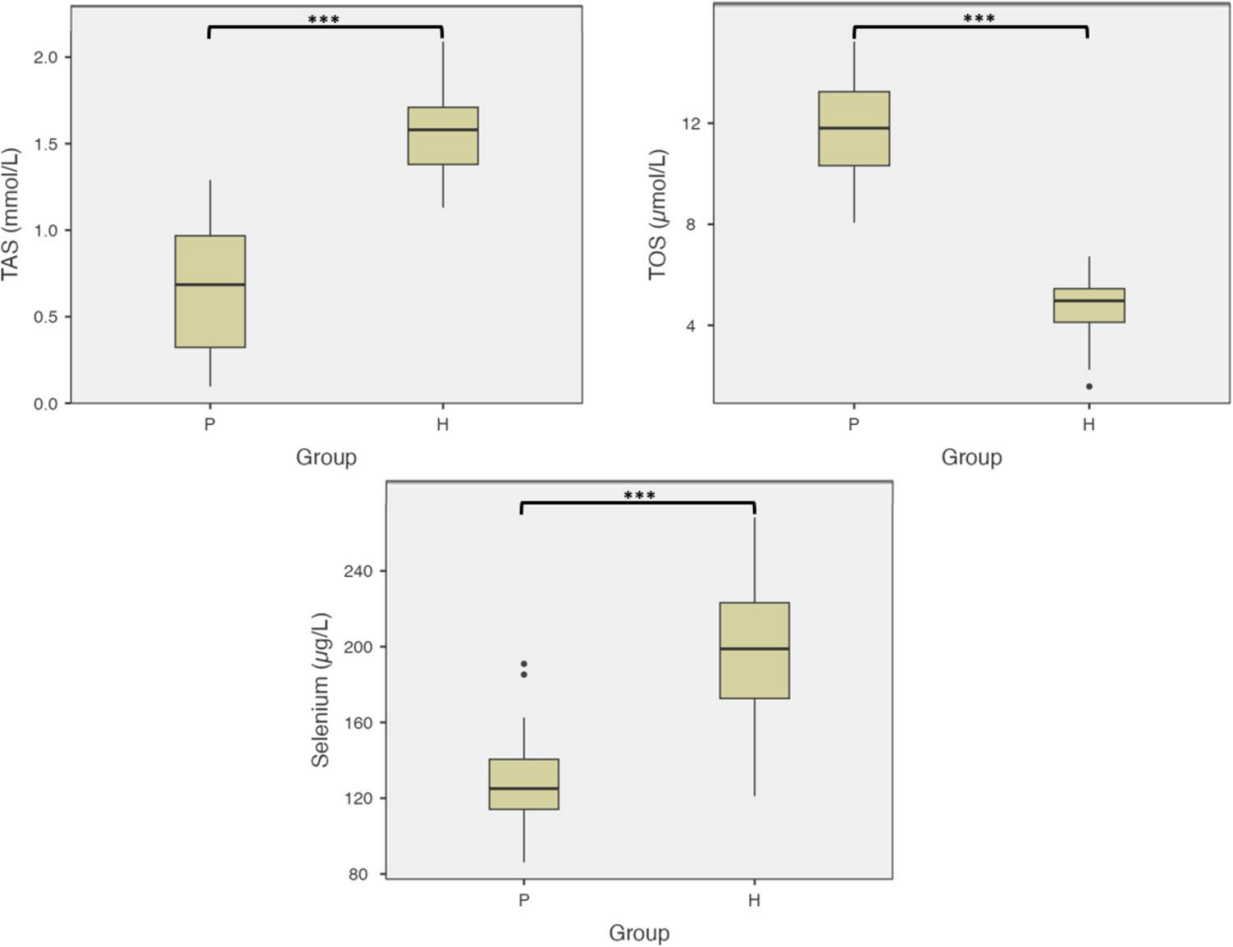
Boxplots of TAS, TOS and selenium levels. TAS and selenium levels were significantly higher in Group H compared to Group P. TOS levels were significantly higher in Group P compared to Group H. *** indicates statistical significance between groups (*p* < 0.001). P: Periodontitis group; H: Periodontally healthy group

A comparison of TOS levels between the study groups revealed significantly higher values in Group P than in Group H (*p* < 0.001). In contrast, the TAS and selenium levels were significantly higher in Group H than in Group P (*p* < 0.001). Group comparisons of the TOS, TAS, and selenium levels are presented in Table 3 and Fig. 3.

**Table 3:** Group comparisons in terms of TOS, TAS, and selenium

	Periodontitis (*N* = 61)	Healthy (*N* = 61)		*p* value
TOS (µmol/L)				
Mean ± sd	11.74 ± 1.92	4.72 ± 1.12	Z = −9.528	< 0.001^*^
Mdn.(min.-max.)	11.80 (8.06–15.23)	4.97 (1.58–6.73)		
TAS (mmol/L)				
Mean ± sd	0.67 ± 0.34	1.57 ± 0.22	Z = −9.462	< 0.001^*^
Mdn.(min.-max.)	0.69(0.10–1.29)	1.58(1.13–2.09)		
Selenium (µg/L)				
Mean ± sd	127.07 ± 21.21	198.47 ± 35.26	t = −13.552	< 0.001^*^
Mdn.(min.-max.)	125.07(86.14–190.92)	198.86(121.10–268.23)		

Values are presented as mean ± standard deviation (sd), median (Mdn.), minimum (min.), maximum (max.)

Z: Mann Whitney U test, t: Independent samples t test

*p < 0,05 and *p*-values less than 0.05 are indicated in bold

### Correlation

The correlations among biochemical parameters were statistically significant (*p* < 0.001). There was a strong negative correlation between the TOS and TAS values, a moderately negative correlation between the TOS value and selenium level, and a moderately positive correlation between the TAS value and selenium level. The relationships between clinical (PI, GI, PD, and CAL) and biochemical (TOS, TAS, and selenium) parameters were also significant (*p* < 0.001). There was a strong positive correlation between the TOS value and clinical parameters, a strong negative correlation between the TAS value and clinical parameters, and a moderate negative correlation between the selenium level and clinical parameters. The correlations between the various parameters are presented in Table 4.

**Table 4:** Correlation of clinical parameters with TOS, TAS, and selenium

		PI	GI	PD	CAL	TOS (ΜMOL/L)	TAS (MMOL/L)	SELENIUM (ΜG/L)
PI	r	—						
	*p*-value	—						
	N	—						
GI	r	0.878	—					
	*p*-value	** < 0.001**	—					
	N	122	—					
PD	r	0.766	0.781	—				
	*p*-value	** < 0.001**	** < 0.001**	—				
	N	122	122	—				
CAL	r	0.773	0.771	0.855	—			
	*p*-value	** < 0.001**	** < 0.001**	** < 0.001**	—			
	N	122	122	122	—			
TOS (µmol/L)	r	0.764	0.776	0.710	0.762	—		
	*p*-value	** < 0.001**	** < 0.001**	** < 0.001**	** < 0.001**	—		
	N	122	122	122	122	—		
TAS (mmol/L)	r	−0.766	−0.743	−0.758	−0.757	−0.763	—	
	*p*-value	** < 0.001**	** < 0.001**	** < 0.001**	** < 0.001**	** < 0.001**	—	
	N	122	122	122	122	122	—	
Selenium (µg/L)	r	−0.649	−0.552	−0.641	−0.630	−0.604	0.625	—
	*p*-value	** < 0.001**	** < 0.001**	** < 0.001**	** < 0.001**	** < 0.001**	** < 0.001**	—
	N	122	122	122	122	122	122	

r: Pearson correlation coefficient

*p*-values less than 0.05 are indicated in bold

*p*-values less than 0.05 are indicated in bold

### Regression Analysis

Due to the presence of multicollinearity and a strong positive correlation between the TOS and TAS values, selenium was included in the regression model. Univariate logistic regression analysis (Table 5) revealed a significant relationship between selenium levels and periodontitis (*p* < 0.001). Furthermore, after adjusting for confounding variables in the model, the selenium level was still strongly associated with periodontitis (*p* < 0.001).

**Table 5:** Logistic regression model for periodontitis

Variable	Crude OR (95% CI)	*p* value	Adjusted OR* (95% CI)	*p* value
Selenium	1.10 (1.06–1.14)	** < 0.001**	1.10 (1.06–1.15)	** < 0.001**
Age	0.85 (0.79–0.92)	** < 0.001**	0.82 (0.72–0.95)	**0.006**
Gender	0.94 (0.46–1.91)	0.86	0.97 (0.24–4.01)	0.97

*Model adjusted for all variables listed

*p*-values less than 0.05 are indicated in bold

### Receiver Operating Characteristic Curves

**Fig. 4:**
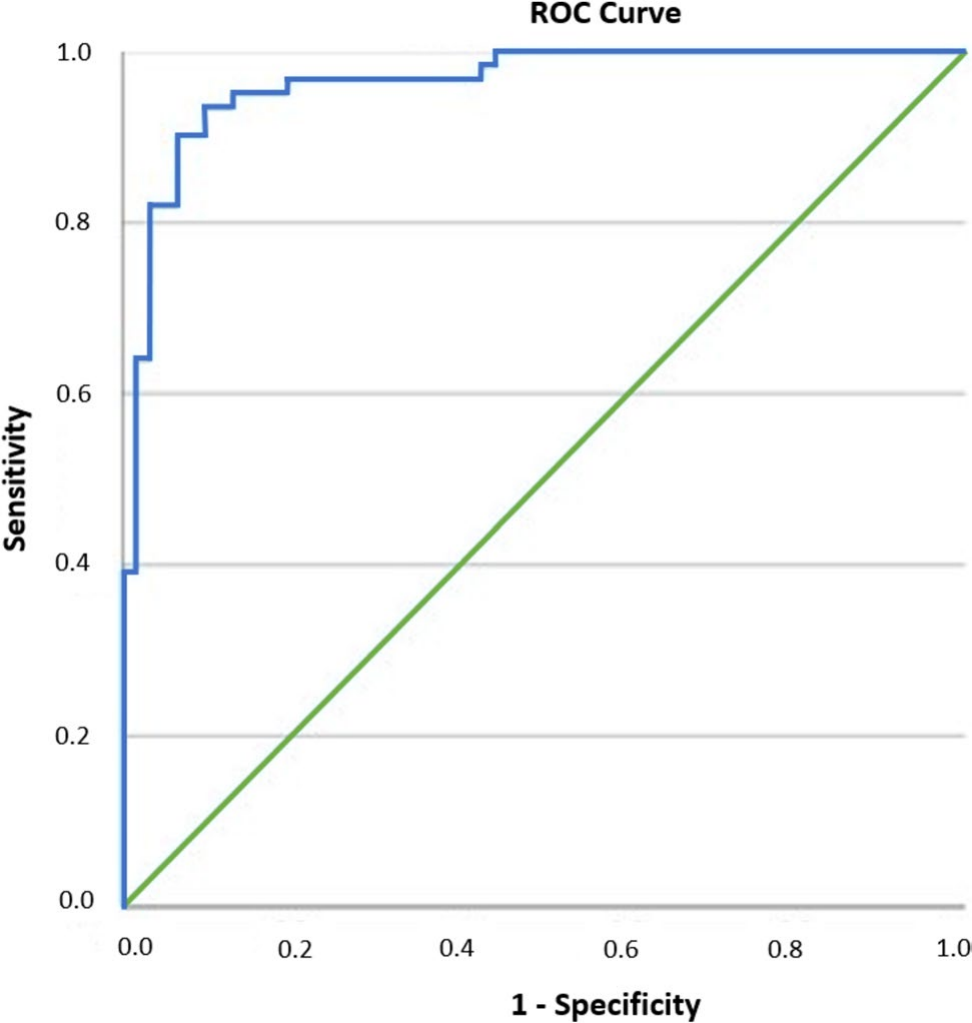
Receiver operator characteristic** (**ROC) curves of selenium levels in predicting for periodontitis

The optimal cut-off selenium level for determining periodontitis risk, identified using receiver operating characteristic (ROC) curve analysis of the case and control groups, was 152.84 μg/L (area under the curve: 0.96, 95% confidence interval [CI]: 0.931–0.993, *p* < 0.018; Fig. 4). The periodontitis risk was found to be higher below this threshold (sensitivity: 90.2%, specificity: 90.2%).

#### ANCOVA

On the basis of the significant group difference in age, ANCOVA was performed to determine the significance of the variables while controlling for age (Table 6). The results indicated that the PI, GI, PD, CAL, TOS, TAS, and selenium levels remained independently significant even after adjusting for age.

**Table 6:** ANCOVA result

		PI		GI		PD		CAL	
		F	p	F	p	F	p	F	p
ANCOVA RESULT	Intercept	6.095	0.204	8.613	0.159	8.858	0.174	1.058	0.476
	Age	0.000	0.996	0.202	0.654	1.332	0.251	1.635	0.203
	Group	243.41	**<.001**	193.38	**<.001**	315.17	**<.001**	464.24	**<.001**
				TOS (µmol/L)		TAS (mmol/L)		SELENIUM (µg/L)	
				F	p	F	p	F	p
ANCOVA RESULT			Intercept	5.156	0.243	6.647	0.194	14.898	0.106
			Age	0.002	0.962	0.348	0.557	0.420	0.518
			Group	500.535	**<.001**	240.153	**<.001**	158.020	**<.001**

*p*-values less than 0.05 are indicated in bold

## Discussion

ROS and their counteracting antioxidants are known to play important roles in chronic inflammatory conditions, such as periodontitis [1, 31, 32]. Low serum antioxidant levels in periodontitis patients are thought to result from the inflammatory process and are considered a risk factor for periodontitis [33]. Several studies have been conducted on the effects of selenium, a potent antioxidant, on periodontitis [23, 34, 35]. The present study contributes to the literature, as it is the only study to evaluate the associations of periodontitis with serum TAS values, TOS values, and selenium levels in healthy individuals. Furthermore, unlike similar studies in the literature, our study provides a comparative assessment specifically targeting stage III and IV periodontitis patients according to the currently employed periodontitis classification system [26].

In our study, TOS and TAS were measured using a novel automatic method developed by Erel, which has shown efficacy in several studies [36, 37]. ICP‒MS, a widely used technique with low detection limits, was used for assessing selenium levels. It can be used to assess both total element and isotope levels. Studies have demonstrated that ICP-MS provides the advantages of precision, speed, reliability, and cost-effectiveness in the analysis of low-concentration trace elements and for routine detection [30].

TOS is a contemporary parameter for detecting tissue damage and oxidative activity caused by FRs. TOS measurement is a practical and reliable method that allows comprehensive assessment, circumventing the need to measure different oxidative species individually [24, 37]. Consistent with previous studies [24, 38, 39], our study also revealed significantly elevated serum TOS levels in periodontitis patients compared with healthy controls.

TAS is a parameter used to comprehensively quantify the total antioxidant capacity instead of measuring the levels of individual antioxidants. This method also has the potential to reveal previously unknown effects of antioxidants [36, 40, 41]. Our study revealed statistically significant differences, with lower serum TAS values in periodontitis patients than in healthy individuals, which is in agreement with the findings of previous studies [24, 25].

Chronic inflammatory diseases may lead to a decrease in antioxidant levels [32]. A deficiency in selenium, a constituent of selenoproteins important for preventing oxidative damage, is considered a risk factor for various diseases [18, 19, 42]. A previous study reported a correlation between periodontitis and deficiencies in micronutrients, including selenium [43]. Selenium and selenoproteins contribute to the pathogenesis of chronic inflammatory diseases, including periodontitis, because of their role in immune regulation, cytokine secretion, immune cell activation, and the regulation of TNF-α produced by macrophages [21]. On the basis of this information, the pathogenesis of diseases characterized by increased oxidative stress markers, such as periodontitis, may be associated with reduced selenium levels.

Thomas et al. conducted a study evaluating serum glutathione, catalase, and selenium levels in periodontitis patients and reported lower serum selenium levels in the periodontitis group than in the healthy group [23]. Similarly, a study focusing on micronutrients by Thomas et al. reported lower selenium levels in periodontitis patients than in healthy individuals [34]. Similarly, our study revealed lower serum selenium levels in the periodontitis group than in the healthy group. In the literature, only one previous study reported an increase in selenium levels in periodontitis patients, attributed to the increased need for selenium to combat the oxidative stress associated with periodontitis [35].

The present study revealed positive correlations between the TOS value, which is an oxidative stress marker, and clinical parameters (PI, GI, PD, and CAL), whereas TAS values and selenium levels were negatively correlated with clinical parameters. These findings suggest a potential relationship between periodontal disease severity and biochemical parameters (TAS, TOS, and selenium). Since the level of ROS increases due to PMNL activation in periodontitis, these correlations further support the associations between periodontal disease severity and biochemical parameters. Additionally, our study revealed significantly lower serum selenium levels in periodontitis patients than in healthy individuals, indicating that selenium levels may serve as a useful biomarker for periodontitis diagnosis.

It has been suggested that serum biomarker analysis may provide valuable information regarding the impact of diet on diseases [33]. Selenium intake is known to be associated with a reduced incidence of oxidative stress and inflammation. Studies have reported positive outcomes of dietary selenium supplementation [43, 44]. Moreover, the World Health Organization’s dietary recommendations for individuals at high risk for chronic diseases such as periodontitis are also in agreement with these studies [45]. Therefore, incorporating selenium into the diet may contribute to the prevention of inflammatory diseases, including periodontitis, and mitigate their destructive effects. However, further randomized clinical trials are needed to evaluate the efficacy and safety of selenium supplementation because of the risk of toxicity.

Our study had several limitations. We could not assess the relationship between selenium levels and dietary habits due to the absence of dietary control in our study. Future studies including variables such as dietary habits, body mass index, physical activity, and lifestyle may yield more robust and comprehensive results. Furthermore, the limited number of studies regarding the threshold serum selenium concentration for diagnosing periodontitis necessitated the evaluation and comparison of healthy individuals and periodontitis patients in our study. As a result, the sample size of our study was small. Despite these limitations, our study demonstrated potential relationships between periodontitis and the levels of TOS, TAS, and selenium.

## Conclusion

In summary, our study revealed significant relationships between periodontitis and the TOS value, the TAS value, and selenium level. Selenium levels were significantly lower in the periodontitis group than in the healthy group. Although our study has certain limitations, the results indicate that the selenium level may serve as a reliable biomarker for evaluating both the activity and severity of periodontal disease. In addition, considering the critical role of selenium in the antioxidant defence system, the evaluation of such trace elements could represent a valuable approach for identifying risk factors associated with the grading of periodontitis. However, further clinical studies are needed to fully elucidate the role of selenium in periodontitis.

## Data Availability

No datasets were generated or analysed during the current study.
